# Percutaneous Coronary Intervention in Patients With Gynecological Cancer: Machine Learning-Augmented Propensity Score Mortality and Cost Analysis for 383,760 Patients

**DOI:** 10.3389/fcvm.2021.793877

**Published:** 2022-02-14

**Authors:** Nicole Thomason, Dominique J. Monlezun, Awad Javaid, Alexandru Filipescu, Efstratios Koutroumpakis, Fisayomi Shobayo, Peter Kim, Juan Lopez-Mattei, Mehmet Cilingiroglu, Gloria Iliescu, Kostas Marmagkiolis, Pedro T. Ramirez, Cezar Iliescu

**Affiliations:** ^1^Division of Cardiology, The University of Texas Health Sciences Center at Houston, Houston, TX, United States; ^2^Department of Cardiology, The University of Texas M.D. Anderson Cancer Center, Houston, TX, United States; ^3^Center for Artificial Intelligence & Health Equities, Global System Analytics & Structures, New Orleans, LA, United States; ^4^Department of Internal Medicine, University of Nevada Las Vegas School of Medicine, Las Vegas, NV, United States; ^5^Department of Internal Medicine, The University of Texas Health Sciences Center at Houston, Houston, TX, United States; ^6^Division of Cardiovascular Disease, University of Arkansas for Medical Sciences, Little Rock, AR, United States; ^7^Department of GynOnc and Reproductive Medicine, The University of Texas Health Sciences Center at Houston, Houston, TX, United States

**Keywords:** gynecologic malignancies, gynecological tumors, PCI, percutaneous coronary intervention, cardio oncology

## Abstract

**Background:**

Despite the growing number of patients with both coronary artery disease and gynecological cancer, there are no nationally representative studies of mortality and cost effectiveness for percutaneous coronary interventions (PCI) and this cancer type.

**Methods:**

Backward propagation neural network machine learning supported and propensity score adjusted multivariable regression was conducted for the above outcomes in this case-control study of the 2016 National Inpatient Sample (NIS), the United States' largest all-payer hospitalized dataset. Regression models were fully adjusted for age, race, income, geographic region, cancer metastases, mortality risk, and the likelihood of undergoing PCI (and also with length of stay [LOS] for cost). Analyses were also adjusted for the complex survey design to produce nationally representative estimates. Centers for Disease Control and Prevention (CDC)-based cost effectiveness ratio (CER) analysis was performed.

**Results:**

Of the 30,195,722 hospitalized patients meeting criteria, 1.27% had gynecological cancer of whom 0.02% underwent PCI including 0.04% with metastases. In propensity score adjusted regression among all patients, the interaction of PCI and gynecological cancer (vs. not having PCI) significantly reduced mortality (OR 0.53, 95%CI 0.36–0.77; *p* = 0.001) while increasing LOS (Beta 1.16 days, 95%CI 0.57–1.75; *p* < 0.001) and total cost (Beta $31,035.46, 95%CI 26758.86–35312.06; *p* < 0.001). Among gynecological cancer patients, mortality was significantly reduced by PCI (OR 0.58, 95%CI 0.39–0.85; *p* = 0.006) and being in East North Central, West North Central, South Atlantic, and Mountain regions (all *p* < 0.03) compared to New England. PCI reduced mortality but not significantly for metastatic patients (OR 0.74, 95%CI 0.32–1.71; *p* = 0.481). Eighteen extra gynecological cancer patients' lives were saved with PCI for a net national cost of $3.18 billion and a CER of $176.50 million per averted death.

**Conclusion:**

This large propensity score analysis suggests that PCI may cost inefficiently reduce mortality for gynecological cancer patients, amid income and geographic disparities in outcomes.

## Introduction

Cardiovascular disease (CVD) and cancer remain the two most common causes of mortality among non-communicable diseases in Western countries (1). The bidirectional relationship between the two, with cancer patients or survivors having a significant burden of CVD and patients with CVD posing an increase in cancer incidence, has become more evident over the last decade and is reflected by the heightened interest in the discipline of cardio-oncology (2, 3). Common risk factors such as tobacco use, poor diet, and chronic inflammatory state are implicated in both disease states (4). Cancer commonly induces a pro-thrombotic state, which can be compounded by side effects of surgical interventions, chemotherapy, radiotherapy, and immunotherapy (3, 5) and trigger cardiovascular events. The recent improvement in overall long-term survival of cancer patients (6), likely related to the progress in cancer therapies, has been paralleled by an increase in the number of percutaneous coronary interventions (PCI) performed in cancer patients (7). Knowing the prevalence of acute coronary syndrome (ACS) in the general population requiring PCI, CVD burden in these cancer patients appears to be vastly underestimated.

Treatment of ACS in cancer patients is challenging, as each type of cancer has a unique clinical presentation and underlying physiology that calls for personalized care. The primary organ site, stage, and presence of metastases are all modifying factors that can influence post-PCI outcomes. Historically this understanding has not been reflected in clinical practice, partly due to the exclusion of patients with cancer from cardiovascular clinical trials and vice versa (8–10). While there is now limited data exploring the overall prognostic impact of cancer on PCI outcomes (11–14), there is no data regarding PCI outcomes in gynecological cancer patients. Reported incidence of gynecologic malignancies in the U.S. is approximately 94,000 cases per year (15), with the most common malignancy being uterine cancer (26.82 cases per 100,000) and the least common vaginal cancer (0.66 per 100,000).

Gynecological cancer patients have special considerations when determining risk for ACS and potential intervention with PCI. Women with endometrial cancer, a population particularly characterized by significant rates of obesity and diabetes mellitus, have been found to have a 1.5-fold increased 10-year risk of CVD when compared to the general population (16). As many as 22% of endometrial cancer patients present at diagnosis with three or more risk factors of coronary artery disease (CAD) (16). Furthermore, death from CVD has been found to be more prevalent in patients with endometrial cancer (17). In women who have undergone debulking procedures for epithelial ovarian carcinoma, the highest risk for hospital readmission perioperatively is a cardiopulmonary event (18). Platinum-based chemotherapeutic agents are frequently utilized for the treatment of ovarian and cervical cancer and are associated with multiple cardiotoxic side effects, with such cardiotoxic drugs as anthracyclines (including doxorubicin and cyclophosphamide) being frequently used for recurrent ovarian cancer (19). While the safety of common cardiovascular interventions such as percutaneous coronary intervention (PCI) in gynecologic cancer patients is not well-described, coronary artery bypass grafting (CABG) is considered a relative contraindication in patients with cancer due to an increased risk of metastatic dissemination during extracorporeal circulation (20). To bridge this knowledge gap, we used a large contemporary national database and examined the outcomes and economics of revascularization procedures in patients with gynecologic malignancies, stratified by specific type of cancer and stage.

## Methods

We defined gynecologic cancer in this analysis as any cancer involving the female reproductive system and further classified it based on specific anatomic location, including cancers of the ovaries, cervix, uterus, vagina, and vulva.

### Data Source

The data source for this study was the 2016 United States (U.S.) National Inpatient Sample (NIS) for hospital discharges, the largest all-payer inpatient dataset in the nation, sponsored by the U.S. Department of Health and Human Services' Agency for Healthcare Research and Quality and maintained within the Healthcare Cost and Utilization Project (HCUP). The NIS currently accounts for approximately 1 in 5 discharges from all community hospitals in the U.S. To reduce sampling bias, the sampling strategy has been modified in the most recent data to produce results more generalizable to all inpatient discharges in the country. In 2016, the NIS data coding adopted the International Classification of Diseases, Tenth Revision, Clinical Modification (ICD-10-CM). Diagnoses of cancer and CVD were maded up to and including the index hospitalization period per patient based on the reported ICD-10. Cardiotoxic oncological treatment both prior and active were not reported in the dataset.

### Study Design

This is the first nationally representative multicenter analysis of inpatient mortality and total cost among all eligible hospitalized adults with CAD by PCI (yes/no) and PCI and cancer (yes/no), including overall and by primary organ site. The 2016 NIS dataset was selected for this study as it is the among latest available datasets and the first to use ICD-10 coding and thus betterreflects current clinical trends in PCI use compared to prior available datasets. Study inclusion criteria was all NIS hospitalizations for adults age 18 years or older during 2016. This study used de-identified data and was conducted according to the ethical principles in the Declaration of Helsinki.

Subjects undergoing PCI were identified by the ICD-10 procedure codes of 00.66 (percutaneous transluminal coronary angioplasty), 36.06 [insertion of non-drug-eluting coronary artery stent(s)], or 36.07 [insertion of drug-eluting coronary artery stent(s)]. ICD-10 diagnosis were used to identify gynecological cancers: C540, C541, C542, C543, C548, C549, C55, D070, Z8542, C530, C531, C538, C539, D060, D061, D067, D069, R87610, R87611, R87612, R87613, R87614, Z8541, Z86001, C561, C562, C569, Z8543, C510, C511, C512, C518, C519, C52, C5700, C5701, C5702, C5710, C5711, C5712, C5720, C5721, C5722, C573, C574, C577, C578, C579, C58, D071, D072, D0730,D0739, R87620, R87621, R87622, R87623, R87624, Z8540, Z8544. ICD-10 codes were used to identify demographics, comorbidities, and outcomes. HCUP tools such as the Clinical Classification Software, which had been used prior to the NIS 2016 dataset for such purposes as classifying cancer (e.g., by primary type and current vs. historical), were not used in this study because they were found by HCUP as a beta version to be unreliable when applied to the 2016 dataset's ICD-10 data.

### Bivariable Statistical Analysis

Descriptive statistics for demographics and comorbidities were performed for the full sample. Comorbidities were selected for analysis (and identified in the dataset by their ICD-10 scores) based on their clinical and/or statistical significance for similar studies in the existing literature. The comorbidities included in this study were diabetes, hypertension, peripheral vascular disease, hyperlipidemia, smoking, obesity, poor diet, stroke, congestive heart failure, cardiac arrest, myocardial infarction, cardiogenic shock, valvular disease, HIV, alcohol abuse, opioid abuse, anemia, chronic obstructive pulmonary disease, coagulopathy, depression, cirrhosis, chronic kidney disease, and malignancy (overall and by primary malignancy type).

Bivariable sub-group analysis was then conducted among gynecological malignancy patients according to the following: (a) inpatient all-cause mortality (yes/no); (b) PCI (yes/no) among the overall sample, stratified by metastases (yes/no) and in subgroup analyses among patients with malignancy; (c) PCI vessel number (multi- vs. single-vessel); (d) malignancy (yes/no) in subgroup analyses among patients who died with non-ST segment elevation myocardial infarction (NSTEMI) and separately among those with ST segment elevation myocardial infarction (STEMI); (e) length of stay by gynecological malignancy type; (f) total cost by gynecological malignancy type. For continuous variables, independent sample *t*-tests were performed to compare means and Wilcoxon rank sum tests were performed for medians. For categorical variables, Pearson chi square tests or Fisher exact tests were performed to compare proportions.

### Regression Statistical Analysis

To optimize the likelihood of validated and replicable results, the performance of the final multivariable regression models in sub-group analysis among gynecological malignancy patients was first assessed by backward propagation neural network machine learning by accuracy and root mean squared error (RMSE) to ensure they were comparable based on an integrated hybrid methodology of traditional statistics reinforced by machine learning (21, 22). Variables found to be statistically significant in the bivariable analysis were included in forward and backward stepwise regression to augment decision-making on which variables should be included in the final multivariable regression models. This regression analysis adjusted for the PCI propensity score was conducted to assess the following outcomes: (a) inpatient all-cause mortality (by logistic) and (b) total hospital costs (by linear, adjusting with the additional variable of total all-cause length of stay) using the predictor of the interaction term between PCI and malignancy (to provide separate estimates of the associations of mortality and PCI, mortality and malignancy, and mortality with PCI and malignancy). The regression models separately assessed these outcomes according to the following major predictors: (a) historical or active malignancy (yes/no), and gynecological malignancy type (uterus, cervical, ovarian, other). Sub-group analysis without propensity score adjustment was conducted separately according to history of CAD (additionally with stratified analysis by ACS and active or prior malignancy), active malignancy, prior malignancy, presenting diagnosis of ACS, NSTEMI, unstable angina, UA), and STEMI. All models adjusted for age, race, income, geographic region, metastases, and mortality risk by diagnosis-related group (DRG). Other variables were excluded based upon the machine learning analysis and diagnostic testing to produce the most clinically and statistically justifiable models.

Next, machine learning-backed propensity score–adjusted multivariable regression was conducted for mortality and controlled for age, race, income, presence of metastases, and mortality risk by diagnosis-related group in addition to the likelihood of undergoing PCI and the NIS weights accounting for the cluster sample data structure. The propensity score was then created for the likelihood of undergoing PCI [the treatment, utilizing the same above variables used in the final regression model to given the double propensity score adjustment method (23–25)], balance was confirmed among blocks, and then the propensity score was included in the final regression models as an adjusted variable. This causal inference approach (propensity score adjustment) was selected because it is a widely accepted methodology to reduce but not eliminate selection bias and the effect of confounding variables. Such competing causal inference approaches as fixed, random, and mixed effects were not appropriate, though these have the added advantage of reducing unobserved variable bias, because the dataset lacked adequate repeated hospitalizations from the same subjects. Propensity score adjustment was used rather than covariate adjustment without the propensity score to enable a more complicated propensity score model (i.e., able to test interactions and higher order terms to produce the best estimated probability of treatment assignment) without risking over-parameterizing while still permitting diagnostic analysis of the final models to be done to confirm superior performance to simple covariate adjustment without the propensity score. Finally, propensity score adjustment rather than competing propensity score techniques was used because of its superior performance in the appropriate context (confirmed by current statistical theory and adequate diagnostic quantitative testing of the final models in cardiovascular studies) (23, 24), and because its inclusion in the final regression models had sufficient performance confirmation the below diagnostic tests.

To modify the final models until optimal performance was achieved, performance was first assessed relative to results from backward propagation neural network machine learning to ensure comparability by root mean squared error and accuracy. Regression model performance was additionally assessed with correlation matrix, area under the curve, Hosmer-Lemeshow goodness-of-fit test, Akaike and Schwarz Bayesian information criterion, variance inflation factor, and tolerance, multicollinearity, and specification error.

The utility of this above hybrid analytic approach, which integrates the traditional statistical method of frequentist-based multivariable regression (supported by propensity score-based causal inference analysis) and supervised learning-based machine learning has been previously demonstrated, as causal inference results which are more familiar to medical science audiences can be confirmed and replicated automatically through machine learning (and thus may accelerate real-time findings on larger high-dimensional datasets as they already increasingly do for other economic sectors outside of medicine), while producing more rapid and accurate results compared to traditional statistics (25–30). An academic physician-data scientist and biostatistician (DJM) confirmed that the final regression models were sufficiently supported by the existing literature and clinical and statistical theory. Fully adjusted regression results were reported with 95% confidence intervals (CIs) with statistical significance set at a 2-tailed *p*-value of < 0.05.

### Cost Effectiveness Analysis

Cost-effectiveness analysis was conducted according to the methodology detailed by the Centers for Disease Control and Prevention (cdc.gov/policy/polaris) and applied to PCI (intervention) vs. medical management alone (comparator): the net cost was calculated as the cost of implementation minuts the averted cost which then produced the ratio of net costs over change in health outcome or the cost-effectiveness ratio (CER), with a negative value in the ratio representing cost savings and a positive value indicating increased cost. The implementation cost was determined by the higher end of the cost of inpatient PCI taken from the National Cardiovacular Registry CathPCI Registry (31) and then multiplied by the number of procedures in the specified sub-group of cardio-oncology patients below in this study's principle dataset (NIS). The averted cost was determined by the 2016 World Bank average life expectancy (worldbank.org/world-development-indicators) minutes the average 2016 NIS age in this study multiplied by the 2016 Quality Adjusted Life Year ($50,000/year/patient) and the cases of mortality averted with the treatment vs. the comparator. The net national cost was calculated as the above implementation cost minus the averted cost. The CER was the above net national cost divided by the number of averted costs by the treatment vs. the comparator.

### Software

Statistical analysis was performed with STATA 14.2 (STATACorp, College Station, Texas, USA), and machine learning analysis was performed with Java 9 (Oracle, Redwood Chores, California, USA).

## Results

### Descriptive Statistics and Bivariable Analysis

Of the 30,195,722 hospitalized patients meeting criteria, 383,760 (1.3%) had gynecological cancer. Among those, mean age was 63.3 years (standard deviation [SD] 15.7), 73.53% were Caucasian, 38.07% had uterine cancer, 29.95% had cervical cancer, 29.51% had ovarian cancer, and 2.47% had other gynecological malignancy (Table 1). Out of the 383,760 patients with gynecological cancer, 7,215 (1.9%) underwent PCI; of those who underwent PCI, 2,875 (39.8%) had active malignancy and 460 (6.4%) had metastases. Significantly patients with gynecological cancer vs. those without it underwent PCI (1.88 vs. 4.04%, *p* < 0.001) even when matched by age and mortality risk as calculated by the NIS according to DRGs (2.35 vs. 5.52%, *p* < 0.001). Among patients receiving PCI, patients with vs. without gynecological were significantly less likely to have CAD (71.56 vs. 78.22%, *p* < 0.001) and presenting STEMI (10.24 vs. 15.09%, *p* < 0.001), but had comparable likelihood of diabetes, hypertension, and presenting NSTEMI.

**Table 1:** Descriptive statistics and bivariable analysis by inpatient mortality (*N* = 383,760 admissions).

**Variables**	**Sample**	**Inpatient mortality**		***P*-value**
		**No**	**Yes**	
		**(373,1695; 97.38%)**	**(10,065; 2.62%)**	
Demographics, No. (%)				
Age, years, mean (SD)	63.31 (15.68)	63.19 (15.71)	67.82 (13.82)	<0.001
Race				
All groups				<0.001
White	73.53	73.65	69.09	
Black	11.89	11.78	16.05	
Hispanic	8.99	9.02	7.89	
Asian	2.60	2.57	3.72	
Native American	0.46	0.46	0.52	
Other	2.54	2.53	2.73	
Non-white	26.47	26.35	30.91	<0.001
Income quartile				0.461
First	28.78	28.74	30.11	
Second	25.63	25.65	24.61	
Third	24.58	24.60	23.90	
Fourth	21.02	21.01	21.38	
Insurance				
Type				<0.001
Commercial	25.24	25.30	23.12	
Medicare	55.41	55.31	58.96	
Medicaid	15.01	15.11	11.38	
VA	1.95	1.89	4.49	
None	2.39	2.39	2.05	
Non-commercial	74.76	74.70	76.88	0.026
Admission, No. (%)				
Non-elective	73.00	72.55	89.44	<0.001
Weekend	18.42	18.26	24.64	<0.001
Medical history				
Diabetes	19.55	19.54	19.72	0.843
Hypertension	59.09	59.10	58.97	0.907
PVD	3.26	3.26	3.43	0.669
HLD	32.25	32.34	29.11	0.002
Obesity	18.70	18.86	12.67	<0.001
Smoking	1.40	1.42	0.60	0.002
Poor diet	0.13	0.13	0.05	0.320
CVA/TIA	3.05	2.95	6.66	<0.001
CHF	4.49	4.45	5.66	0.010
HFrEF	1.53	1.51	2.14	0.025
Exacerbation	4.15	4.08	6.51	<0.001
Cardiac Arrest	0.51	0.15	13.91	<0.001
Myocardial Infarction	1.92	1.82	5.86	<0.001
STEMI	0.31	0.26	2.19	<0.001
NSTEMI/UA	1.62	1.56	3.78	<0.001
Cardiogenic shock	0.17	0.11	2.48	<0.001
Valvular disease	4.83	4.82	5.02	0.686
HIV	0.32	0.32	0.20	0.335
Alcohol abuse	2.01	2.02	1.69	0.303
Opioid abuse	1.52	1.55	0.60	0.001
Anemia	29.90	29.61	40.64	<0.001
COPD	15.27	15.26	15.45	0.819
Coagulation disorder	6.93	6.62	18.48	<0.001
Depression	15.87	16.01	10.73	<0.001
Cirrhosis	1.71	1.68	2.98	<0.001

*SD, standard deviation; VA, Veteran Affairs; PVD, peripheral vascular disease; HLD, hyperlipidemia; CVA, cerebrovascular disease; TIA, transient ischemia attack; CHF, congestive heart failure; HFrEF, heart failure with reduced ejection fraction; STEMI, ST segment elevation myocardial infarction, NSTEMI, non-ST segment elevation myocardial infarction; UA, unstable angina; HIV, human immunodeficiency virus; COPD, chronic obstructive pulmonary disease; CKD, chronic kidney disease; PCI, percutaneous coronary intervention; CABG, coronary artery bypass graft*.

A total of 794,147 (2.6%) deaths were recorded, out of which 20,807 (2.6%) were from gynecological malignancy (Table 1). Patients with gynecological cancer had significantly lower mortality when compared to non-gynecological cancer patients (2.30 vs. 4.54%, *p* = 0.004). Furthermore, in patients with gynecological malignancy, mortality (yes/no) was significantly lower for Caucasian (69.09 vs. 73.65%) but higher for African American patients (16.1 vs. 11.8%) (both *p* < 0.001) and those with metastases (54.5 vs. 22.6%, *p* < 0.001).

Among patients with gynecological malignancy, the median all-cause length of stay (LOS) was 3 days (range 2–6, *p* < 0.001) and median cost of hospitalization in U.S. dollars was 34,657 (18,894–62,952; *p* < 0.001). The highest mortality (yes/no) percentage was ovarian vs. non-ovarian gynecoloical malignancy (0.60 vs. 0.37%) followed by uterine vs. non-uterine (0.59 vs. 0.48%) (Table 2). The longest mean LOS was ovarian cancer (5.39 days [SD 5.57]), followed by other gynecological malignancy (5.25 days [SD 7.87]), and the most expensive total hospitalization cost was ovarian (USD 56,708 [SD 72440.59]) followed by uterine (53907.51 [SD 69559.61]).

**Table 2:** Summary bivariable outcome results by malignancy (*N* = 383,760 admissions).

**Malignancy**	**Outcomes**			
	Mortality, No. (%)^*^		LOS, days, mean (SD)^**^	Cost, USD, mean (SD)^**^
	**No**	**Yes**		
Gynecological	1.27	1.52	5.03 (5.71)	52925.20 (69153.44)
Uterus	0.48	0.59	4.99 (5.57)	53907.51 (69559.61)
Cervix	0.38	0.30	4.74 (5.51)	48644.10 (64795.88)
Ovarian	0.37	0.60	5.39 (5.57)	56708.13 (72440.59)
Other	0.09	0.07	5.25 (7.87)	52326.71 (67357.82)

*LOS, length of stay; SD, standard deviation; USD, US dollars;*

* *p < 0.05 for mortality (yes vs. no);*

** *p < 0.05 for malignancy (yes/no)*.

### Multivariable Regression

In propensity score-adjusted regression among all patients, the interaction of PCI and gynecological cancer (vs. not having PCI) was associated with significantly reduced mortality (OR 0.53, 95%CI 0.36–0.77; *p* = 0.001; marginal effects likelihood: −0.87%). Among gynecological cancer patients, mortality was similarly significantly reduced by PCI (OR 0.58, 95%CI 0.39–0.86; *p* = 0.007) as well as hospitalization in East North Central, West North Central, South Atlantic, and Mountain regions (all *p* < 0.05) compared to New England. PCI reduced mortality but not significantly for patients with metastatic cancer (OR 0.74, 95%CI 0.31–1.75; *p* = 0.493) (Table 3). There were no significant racial or income disparities.

**Table 3:** Machine learning–augmented propensity score adjusted multivariable regression of inpatient mortality among gynecological malignancy patients (*N* = 383,760 admissions).

**Variable**	**OR (95% CI; *P*-value)**
Age by 10 years	1.00 (0.99–1.00; *p* = 0.136)
Non-white race	**1.22 (1.08**–**1.36;** ***p*** **=** **0.001)**
Region	
Mid-Atlantic	0.95 (0.73–1.24; *p* = 0.715)
East North Central	**0.73 (0.56**–**0.95;** ***p*** **=** **0.021)**
West North Central	**0.71 (0.50**–**0.99;** ***p*** **=** **0.044)**
South Atlantic	**0.75 (0.57**–**0.98;** ***p*** **=** **0.038)**
East South Central	1.04 (0.73–1.48; *p* = 0.830)
West South Central	1.03 (0.76–1.36; *p* = 0.851)
Mountain	**0.61 (0.42**–**0.88;** ***p*** **=** **0.008)**
Pacific	0.99 (0.76–1.29; *p* = 0.935)
Zip code income	
1st quartile	Reference
2nd quartile	0.94 (0.82–1.08; *p* = 0.416)
3rd quartile	0.89 (0.78–1.03; *p* = 0.124)
4th quartile	0.87 (0.75–1.02; *p* = 0.086)
PCI	**0.58 (0.39**–**0.86;** ***p*** **=** **0.007)**
Malignancy	
Metastases	**2.03 (1.84**–**2.24;** ***p*** **<** **0.001)**
Mortality risk by DRG	**7.12 (6.54**–**7.75;** ***p*** **<** **0.001)**

*OR, odds ratio; CI, confidence interval; PCI, percutaneous coronary intervention; DRG, diagnosis-related group. The bold values are statistically significant*.

**Figure 1:**
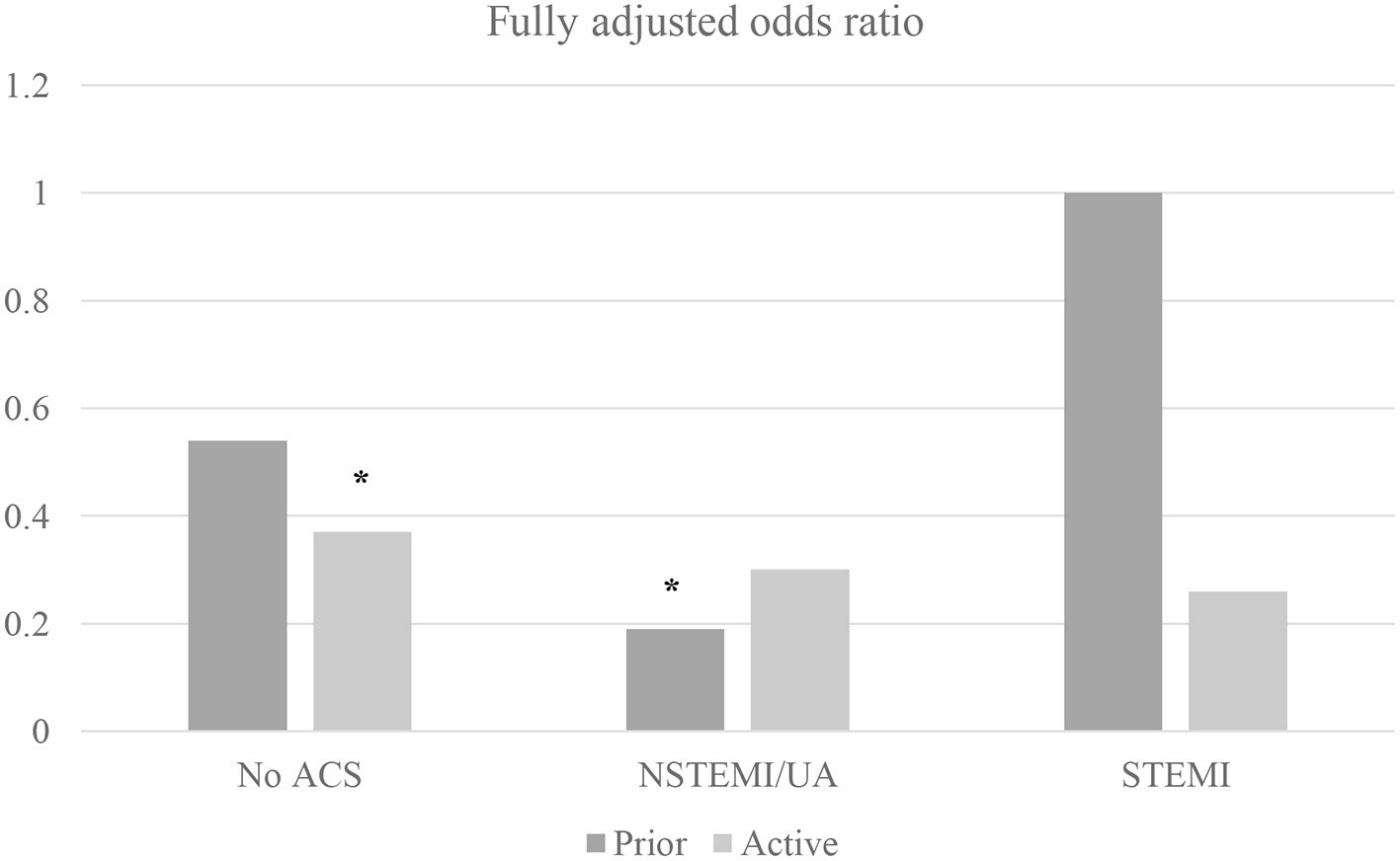
Machine learning–augmented propensity score adjusted multivariable regression of inpatient mortality among gynecological malignancy patients (*N* = 383,760 admissions). Multivariable regression fully adjusted for age, race, income, metastases, and mortality risk by Diagnosis Related Group; NSTEMI/UA, non-ST elevation myocardial infarction/unstable angina; STEMI, ST-elevation myocardial infarction; *p < 0.05.

**Figure 2:**
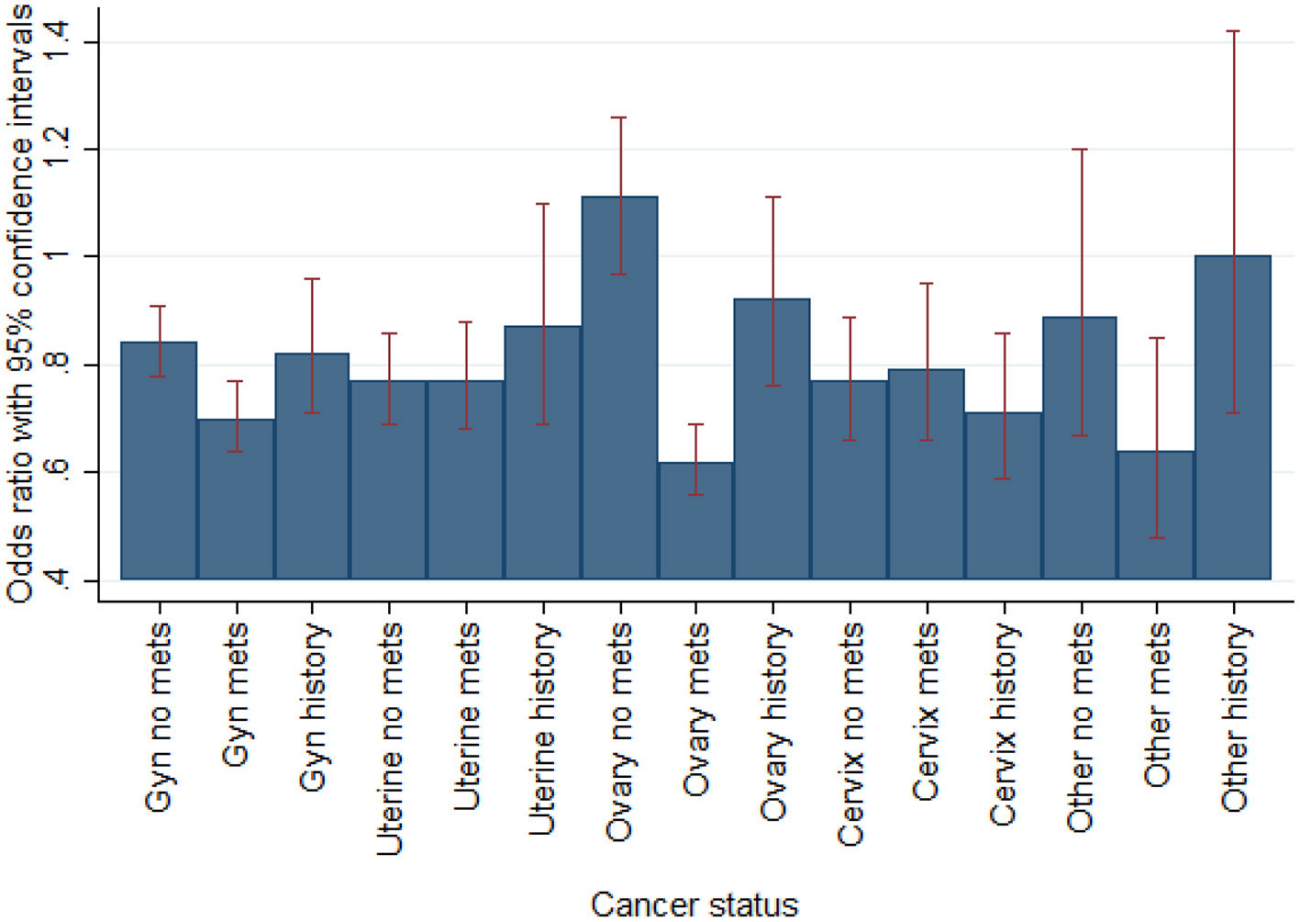
Multivariable regression of mortality by gynecological oncology status vs. no cancer (*N* = 383,760 admissions). Fully adjusted for age, race, income, region, PCI, PCI likelihood, and NIS-calculated mortality risk by DRG.

In sub-group analysis by individual gynecological malignancy type, PCI significantly decreased all-cause mortality for uterine cancer (OR 0.49, 95% CI 0.25–0.96; *p* = 0.038) but not ovarian, cervix, or other. In sub-group analysis by ACS (including separately NSTEMI/UA vs. STEMI) and active malignancy (yes/no) among gynecological malignancy patients, PCI reduced mortality for all sub-groups but only significantly for patients with non-ACS active malignancy patients (OR 0.37, 95% CI 0.15–0.89; *p* = 0.027) and NSTEMI/UA prior malignancy patients (OR 0.19, 95%CI 0.05–0.72; *p* = 0.014) (Figure 1). In sub-group analysis by gynecology cancer by primary organ site and cancer status (without metastasis, with metastasis, and historical diagnosis all vs. no cancer), the highest mortality reductions with PCI were for patients with ovarian metastasis (Figure 2).

### Cost Effectiveness

In propensity score adjusted regression among all patients, the interaction of PCI and gynecological cancer (vs. not having PCI) significantly increased LOS (Beta 1.16 days, 95% CI 0.57–1.75; *p* < 0.001) and total cost of stay (Beta $31035.46, 95% CI 26758.86–35312.06; *p* < 0.001). Of the 7,215 gynecological cancer patients who underwent inpatient PCI, 0.25% or 18 extra gynecological cancer patients' lives were saved with PCI for a net national cost of $3.18 billion and a cost effectiveness ratio (CER) of $176.50 million per averted death.

## Discussion

Our study demonstrated that inpatient PCI can be safely performed in patients with gynecological cancer, including those with metastatic disease, albeit with increased cost and length of stay amid significant geographic disparities in mortality. This is the first known nationally representative, comprehensive machine learning-augmented, propensity score analysis of mortality and cost for patients with gynecological cancer vs. non-gynecological cancer patients in terms of PCI vs. medical management (including overall and by ACS).

Our analysis reveals that PCI does not increase mortality in patients with gynecologic cancer, regardless of the unique risks in this population. When analyzed by specific type of malignancy, PCI significantly reduced mortality for uterine cancer, while ovarian, cervical, and other gynecologic cancers had a non-statistically significant reduction in mortality. This may at least be in part because patients with uterine cancer in contrast to the other gynecological cancers in this dataset had greater CVD risk factors (i.e. older with higher prevalence of hypertension and diabetes) and thus may be positioned to best benefit from PCI. The lack of increased mortality rate across all cancer types is likely not just statistical in nature and could suggest that routine/standard of care PCI if applied to this patient population would not translate in a significant increase in mortality.

Furthermore, this analysis shows that even when patients with gynecological vs. non-gynecological cancer have comparable age and mortality risk, they undergo PCI significantly less than patients without this cancer type, suggesting that inpatient PCI may be withheld from these patients (further research is required to clarify the reasons why which likely are multifactorial and can include lower clinical suspicion or more non-specific symptoms for CVD given typically younger age and less CVD risk factors). This finding was consistent across a wide range of age and mortality risk groups. While PCI may be offered less to cancer patients due to concerns of safety and efficacy, previous literature indicates that PCI is safe and beneficial in such population (11–14), and our real-world analysis shows PCI is safe to perform in gynecological cancer patients as well. The results presented here should promote the inclusion of patients with gynecological cancer undergoing cancer treatment and with acceptable medium- and long-term survival (least 6 months and preferably 1 year expected >50% survival) in future cardiovascular trials and encourage physicians to more frequently utilize PCI in this patient population.

Other factors worth considering in future analyses are the type of stent used and medication used in gynecologic cancer patients. Standard balloon angioplasty or percutaneous old balloon angioplasty (POBA) has been shown to have overall worse outcomes compared to drug-eluting stents in the general population, and was considered a possible option in gynecologic cancer patients as the reduced duration of aspirin and Plavix or dual antiplatelet therapy (DAPT) with POBA may be beneficial to patients with an increased bleeding risk (32). Evolution of stent platforms, polymers and eluting medications over the last decade has translated in an abbreviated DAPT course, for certain indications (stable angina, abnormal stress test) several stent have been approved for 1–3 months of DAPT. Patients with metastatic disease would require additional stratification that impacts decision making in these complex clinical challenges.

Our results should be interpreted with caution in the context their limitations, which include a non-randomized design with administrative data limited to inpatient variables without longitudinal individual follow-up data, particularly 3-month and 12-month mortality which can affect cost-effectiveness analysis. This study sought to overcome such limitations on its external and internal validity by utilizing multicenter nationally representative data with robust causal inference analysis to allow for the most reliable and reproducible results possible for this nuanced clinical topic.

## Conclusion

This study provides evidence that the clinical benefit of PCI may be safely extended to gynecological cancer patients, albeit with an increase in cost. There is also evidence of mortality disparity by geography and PCI underutilization in gynecological cancer patients despite clinical indication. This first known granular sub-group analysis by malignancy type, and active vs. prior cancer status suggests PCI significantly decreases mortality by type of gynecological cancer.

## Data Availability Statement

The original contributions presented in the study are included in the article/supplementary material, further inquiries can be directed to the corresponding author/s.

## Author Contributions

All authors listed have made a substantial, direct, and intellectual contribution to the work and approved it for publication.

## Conflict of Interest

The authors declare that the research was conducted in the absence of any commercial or financial relationships that could be construed as a potential conflict of interest.

## Publisher's Note

All claims expressed in this article are solely those of the authors and do not necessarily represent those of their affiliated organizations, or those of the publisher, the editors and the reviewers. Any product that may be evaluated in this article, or claim that may be made by its manufacturer, is not guaranteed or endorsed by the publisher.
